# The role of TLR7 agonists in modulating COVID-19 severity in subjects with loss-of-function TLR7 variants

**DOI:** 10.1038/s41598-023-40114-8

**Published:** 2023-08-11

**Authors:** Shaik Mohammad Naushad, Gowtham Mandadapu, Mekala Janaki Ramaiah, Fahad N. Almajhdi, Tajamul Hussain

**Affiliations:** 1 Yoda LifeLine Diagnostics Pvt Ltd, 6-3-862/A, Lal Bungalow Add on, Ameerpet, Hyderabad, 500016 India; 2 Devansh Lab Werks, 234 Aquarius Dr, Homewood, AL 35209 USA; 3 K L University, Green Fields, Vaddeswaram, Andhra Pradesh 522502 India; 4 COVID-19 Virus Research Chair, Department of Botany and Microbiology, College of Science, King Saud University, 11451 Riyadh, Saudi Arabia; 5 Center of Excellence in Biotechnology Research, College of Science, King Saud University, 11451 Riyadh, Saudi Arabia

**Keywords:** Computational biology and bioinformatics, Drug discovery, Genetics, Immunology, Microbiology

## Abstract

We investigate the mechanism associated with the severity of COVID-19 in men with TLR7 mutation. Men with loss-of-function (LOF) mutations in TLR7 had severe COVID-19. LOF mutations in TLR7 increased the risk of critical COVID by 16.00-fold (95% confidence interval 2.40–106.73). The deleterious mutations affect the binding of SARS-CoV2 RNA (− 328.66 ± 26.03 vs. − 354.08 ± 27.70, *p* = 0.03) and MYD88 (β: 40.279, *p* = 0.003) to TLR7 resulting in the disruption of TLR7-MyD88-TIRAP complex. In certain hypofunctional variants and all neutral/benign variants, there is no disruption of TLR7-MyD88-TIRAP complex and four TLR7 agonists showed binding affinity comparable to that of wild protein. N-acetylcysteine (NAC) also showed a higher binding affinity for the LOF variants (*p* = 0.03). To conclude, TLR7 LOF mutations increase the risk of critical COVID-19 due to loss of viral RNA sensing ability and disrupted MyD88 signaling. Majority of hypofunctional and neutral variants of TLR7 are capable of carrying MyD88 signaling by binding to different TLR7 agonists and NAC.

## Introduction

The COVID-19 fatality ratio showed a linear increase among individuals > 30 years^1^. Men had more severe COVID-19 and a 2.4-fold increased mortality rate than women^2^. Comorbidities further increase the risk^3^. These studies emphasize the possible association of X-linked genetic variants with the severity of COVID-19. Pattern recognition receptors, TLR signaling regulators, and immune-related genes influenced the severity of COVID-19^4^.

Loss-of-function (LOF) mutations in TLR7 led to critical COVID-19 in men. This is due to impaired synthesis of type I interferon by plasmacytoid dendritic cells. LOF mutations lead to severe COVID-19, which requires mechanical ventilation in four men^6^. The imiquimod TLR7 agonist did not restore the impaired type I and II IFN signaling^6,7^. In a cohort of patients with severe COVID-19, 2.1% of men had LOF mutations that caused reduced expression of TLR7^8^. The TLR7 rs3853839 mutation showed an association with COVID-19 severity with altered ferritin, C-reactive protein (CRP), interleukin (IL)-6, and D dimer^9^. The TLR7 LOF mutations showed a 4.53-fold increase in the risk of severity of COVID-19 in an exome study^10^. RNA sequencing revealed profound impairment of the TLR7 pathway in LOF variants^11^. Hypofunctional and hypomorphic variants also exhibited impaired IFN-gamma regulation^11^.

The serine protease 2 transmembrane protein (TMPRSS2) activates the spike protein of SARS-CoV-2, thus allowing the internalization of the virus particles^12,13^. Viral RNA undergoes replication in the host cytoplasm or enters the endosome. Cellular sensors recognize the viral RNA and elicit the innate immune response. Cytoplasmic RIG-I and MDA5 are the primary sensors in the lung epithelium^14–16^. Plasmacytoid dendritic cells (pDCs) and B cells express endosomal TLR7. It senses the viral genome and recruits MyD88 to carry out a cascade of downstream signaling^17^

The usage of TLR7 agonists to stimulate innate and acquired immunity was effective against COVID-19. TLR7 agonists induce the Th1 antiviral response and are effective as vaccine adjuvants against COVID-19. Besides, they exhibit anti-inflammatory, broncho- and vaso-dilatory functions^18^. Imiquimod induces innate and acquired immunity during the early phases of infections^19^. Nevertheless, its utility in the later phases of infection is questionable as it causes a cytokine storm^20^. Imidazoquinoline conjugated with cholesterol-polyethylene glycol is another adjuvant capable of generating neutralizing antibodies^21^.

Here, we explore the association of TLR7 LOF mutations with the severity of COVID-19. Further, we studied the impact of these mutations on the SARS CoV-2 RNA sensing ability of TLR7. We also explore the impact of the altered viral RNA sensing ability of TLR7 on MyD88 or agonist binding. We also investigated ways to bypass the effect of LOF mutations on TLR7.

## Materials and methods

### Data collection

We analyse clinical and biochemical data from all original studies retrieved by searching the PubMed, Medline, and Google Scholar databases using the following keywords: TLR7, COVID-19, and disease severity. We classified the cases into mild, moderate, severe, and critical. Furthermore, we have searched our internal whole exome data of 1213 subjects to determine the presence of TLR7 pathogenic variants of TLR7 in our population.

### Mutation mapping

We map all TLR7 mutations using the cBioportal (https://www.cbioportal.org/mutation_mapper). This mapping helped in understanding domain-wise harboring of mutations.

### In silico modeling

Sequence-related information retrieved from UniProtKB (ID: Q9NYK1, TLR7_Human). The cryo-electron microscopic structure of human TLR7 in complex with UNC93B1 (PDB ID: 7CYN) was used as a template. We have modeled all TLR7 variants using the Phyre 2 module (http://www.sbg.bio.ic.ac.uk/phyre2).

### Pathogenicity scoring

We assessed the pathogenicity of reported TLR7 variants using various modules. The SIFT, Provean, and SNAP2 modules determined the pathogenicity of missense variants. ENTPRISE-X module determined the pathogenicity of frameshift and nonsense mutations.

We classified mutations as deleterious if the SIFT score is < 0.05, Provean Score < − 2.5, and the SNAP2 score is > 50.

### Position-Specific Evolutionary Preservation (PSEP) time

The mutations that occur in highly conserved regions are also considered deleterious. We measured PSEP time using the Panther module. The functional impact of the nonsynonymous mutation is proportional to the PSEP time. When the PSEP time > 450 my, the mutation is probably damaging. If the PSEP time is between 200 and 450 my, the mutation is possibly damaging. If the PSEP time < 200 my, the mutation is probably benign.

### Thermal stability assessment

Mutations that cause a decrease in thermal stability are also considered damaging. We have used the crystal structure of TLR7 (PDB: 7CYN) as a reference to assess the stability of the variants. CUPSAT determines protein stability on amino acid atomic potentials and torsion angles.

### Stability changes upon mutations

Gibbs free-energy changes induced by mutations are evaluated using the PremPs module as a second measure of protein stability.

### Nucleic acid binding ability

The acquired or innate immune response elicited by TLR7 depends on its ability to detect viral RNA. Hence, we have employed electrostatic potentials to predict the nucleic acid binding sites of TLR7. Using the SARS CoV-2 Chain T RNA (5′R(P*UP*UP*CP*AP*UP*AP*AP*CP*UP*UP*AP*A)-3′) as a reference, explored viral RNA sensing ability of different TLR7 variants. We have used two ssRNA motifs, i.e. 5′-UGCUGUUGUGUGUU-3′ and 5′-GUGUGUGUGUUCUGUUAUU-3′previously reported to induce TLR-MyD88 signaling after COVID-19 infection and docked these variants with the reported TLR7 variants and demonstrated the presence of two RNA binding sites of RNA. This HDock analysis used a hybrid algorithm of template-based and ab initio docking. We used Gibbs free energy change (ΔG) as a measure of binding affinity. In a spontaneous process, protein–ligand binding occurs only when ΔG value of the system is negative in the equilibrium state at constant temperature and pressure. The magnitude of the negative ΔG determines the stability of protein–ligand complex and alternatively indicates the binding affinity of a ligand to a given protein.

### Molecular docking

We assessed whether TLR7 agonists or N-acetyl cysteine could bind to TLR7 variants. The CB- Dock module helped dock imiquimod, Resiquimod, Gardequimod, Vesatolimod and N-acetylcysteine to different variants of TLR7.

#### Protein–protein interactions and network analysis

We used the LZerD webserver to evaluate changes in TLR7-Myd88-TIRAP interactions as a result of mutations in TLR7. This protein docking program takes two or more individual 3D protein structures, which are either experimentally solved or computationally modeled, and outputs a series of probable complex structures.

The cascade events triggered by the viral sensing ability of TLR7 were elucidated through Protein–Protein interaction and network analysis using the Inbio-discover module (https://inbio-discover.com/).

#### Statistical analysis

We used the Student’s t test and analysis of variance (ANOVA) for continuous variables. Student t-tests evaluated differences in pathogenic scores between severe and critical COVID-19. Analysis of variance (ANOVA) evaluated binding affinity changes across different TLR7 functional classes. Fisher’s exact test assessed the severity of COVID-19 with deleterious TLR7 mutations. Pates form the basis for this categorization. All statistical associations are considered significant when *p* < 0.05.

## Results and discussion

We have compiled the data from four published studies i.e.^5–8^ and performed a meta-analysis to determine the demographic and clinical characteristics of critical COVID-19 vs. severe COVD-19 cases along with laboratory findings, TLR7-related genetic and expression studies. These studies included hypofunctional and neutral variants also along with loss-of-function mutations.

In parallel, we have evaluated TLR7 variants using Global screening array in 13 men who had recovered from mild to moderate COVID without the need for hospitalization. We have identified TLR7 variants in 8 of them. Two intronic variants i.e., rs5741880 and rs179010 and one 3′-UTR variant i.e.rs3853839 were observed. None of them had rs179008 variant. (Table 1) In addition, we have searched whole exome sequencing data of 1213 subjects and find none of the loss-of-function or hypofunctional TLR7 variants reported earlier in the four published studies. We have observed TLR7 Q11P polymorphic variant in women with a minor allele frequency of 5.4%. Out of the 267 women examined, 10% were heterozygous and 0.7% were homozygous mutant. In silico studies are not possible in the identified variants as there are not coding variants. We performed in silico analyses only on the published coding variants and correlated with these experimental findings.

**Table 1:** Distribution of four TLR7 polymorphic variants in 13 men who had mild to moderate COVID.

Variant	Location	Wild	Variant	CADD score
rs5741880 G > T	Intronic	10	3	2.163
rs179010 T > C	Intronic	8	5	2.985
rs3853839 G > C	3′-UTR	10	3	0.306
rs179008 A > C	Coding	13	0	13.26

The primary objective of this study was to establish an association of pathogenicity scores with TLR7 activity/expression. The experimental data from four publications showed positive association between TLR7 expression and its activity (r = 0.754, *p* < 0.001). Both expression (r = − 0.719, *p* < 0.001) and activity (r = − 0.701, *p* < 0.001) of TLR7 are inversely associated with SNAP2 score suggesting that mutations with higher SNAP2 score are associated with loss-of-function. This further substantiated by inverse association of CADD scores with both expression (r = − 0.618, *p* < 0.001) and activity of TLR7 (r = − 0.731, *p* < 0.001). This establishes the association of pathogenicity scores with TLR7 activity/expression (Table 2).

**Table 2:** To establish association between pathogenicity scores and TLR7 activity/expression.

Pathogenicity score	NF-KB luciferase activity		TLR7 expression	
	R value	*p*-value	R value	*p*-value
SIFT	0.071	0.706	0.355	0.05
Provean	0.575	0.001	0.623	< 0.001
PSEP time	− 0.395	0.04	− 0.359	0.07
SNAP2	− 0.701	< 0.001	− 0.719	< 0.001
CADD score	− 0.731	< 0.001	− 0.618	< 0.001

R: Correlation coefficient.

ANOVA analysis of CADD scores segregated based on functionality revealed higher CADD scores in Loss-of-function and hypofunctional variants (24.91 ± 3.71 and 21.89 ± 4.16) than the neutral variants (16.39 ± 4.85, F: 9.96, *p* < 0.001). In the current study, the CADD scores of variants in mild to moderate COVID-19 were < 3.0 while those reported in severe and critical COVID had scores ranging from 11.88 to 32 suggesting the positive association of CADD scores with COVID-19 severity.

TLR7 Q11P observed in women is a benign variant as per the SIFT, Provean and CADD scores. Its PSEP time was 220 my and SNAP2 score was 66 suggesting a possibly damaging effect. Our findings were consistent with a recent study that showed null association of this variant with COVID-19 severity^22^. A recent study demonstrated that substitution at this position occurs within the N-terminal signal peptide of TLR7, which determines the cotranslational translocation of the nascent protein to the endoplasmic reticulum. Signal peptide function will be more favorable towards the wild allele^23^.

**Figure 1:**
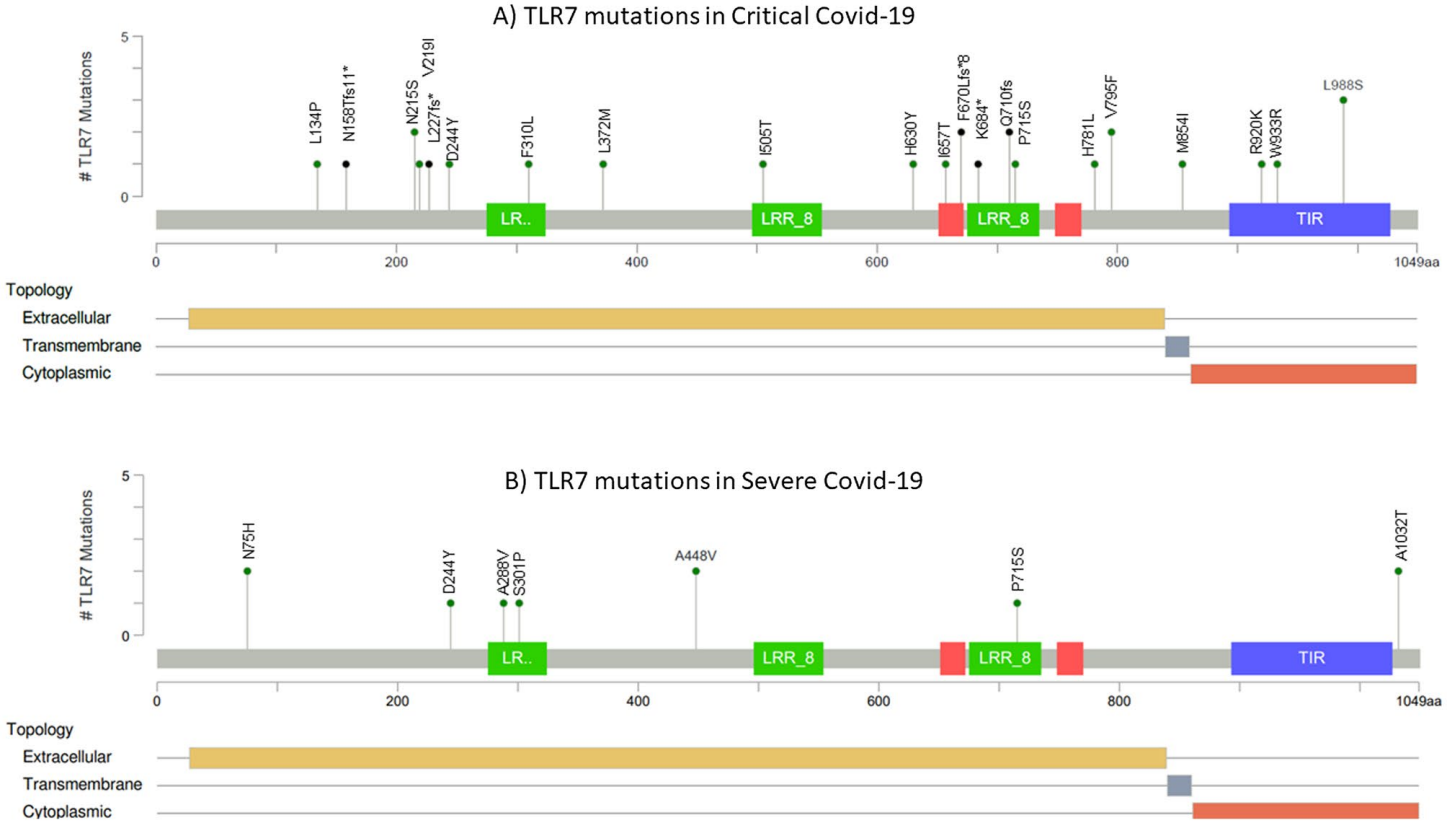
TLR7 mutations reported in COVID-19 critical vs severe cases. (**A**) TLR7 mutations reported in critical COVID-19 cases. (**B**) TLR7 mutations reported in severe COVID-19 cases.

The second objective was to test the utility of the TLR7 pathogenicity scores in determining the severity of COVID-19. Among the different pathogenicity scores evaluated, the PSEP time showed a statistically significant association with the severity of COVID-19. TLR7 mutations in highly conserved regions resulted in critical COVID-19 in men (PSEP time: 440.15 ± 202.83 vs. 280.70 ± 13,091 my, *p* = 0.03). The evaluation of demographic and clinical characteristics in the published cases showed an association of TLR7 mutations with critical COVID-19 in younger men (33.68 ± 16.94 vs. 53.25 ± 11.08 yr, *p* = 0.005). The cases with critical COVID-19 had prolonged ICU stay with an average of 15.38 days, while none of the severe COVID-19 required ICU stay. The requirement for oxygen therapy was 12.22-folds in Critical COVID-19 cases compared to severe COVID-19. About 76% of the cases with critical COVID-19 required intubation, while none of the cases with severe COVID-19 required intubation. Although CRP levels were higher in critical cases of COVID-19, no statistical significance was obtained. Hemoglobin levels and platelet count were not affected based on COVID-19 severity. However, the leukocyte count increased (9664 ± 4329 vs. 3495 ± 710 cells/mm^3^). Although not statistically significant, lymphopenia was observed in critical COVID-19 cases (1436 ± 914 vs. 2553 ± 1158 cells/mm3, *p* = 0.07). NF-KB luciferase activity is significantly lower in critical COVID-19 compared to severe COVID-19 (9.13 ± 24.03 vs. 36.70 ± 44.48, *p* = 0.03), justifying the link of TLR7 pathogenicity scores with critical COVID-19. However, TLR7 expression did not show a statistically significant association with COVID-19 severity. Immunoglobulin profile showed no statistically significant association with COVID-19 severity (Table 3). L134P, N158Tfs11*, N215S, V219I, L227fs*, D244Y, F310L, L372M, I505T, H630Y, I657T, F670Lfs*8, K684*, Q710fs, P715S, H781L, V795F, M854I, R920K, W933R and L988S are the TLR7 mutations observed in critical COVID-19 cases. N75H, D244Y, A288V, S301P, A448V, P715S, and A1032T are the mutations observed in cases of severe COVID-19 (Fig. 1).

**Table 3:** Association of demographic and clinical details according to COVID-19 severity.

Variable	Critical COVID (n = 27)	Severe COVID (n = 10)	t-value	*p*-value
Age (yr)	33.68 ± 16.94	53.25 ± 11.08	3.05	0.005
Mortality	3/25	0/8		0.84
Hospital stay (days)	27.74 ± 25.89	5.25 ± 3.77	1.70	0.10
ICU stay	15.38 ± 19.42	0.00	2.22	0.03
Oxygen therapy	22/25	3/8	12.22 (1.88–79.45)	0.02
Intubation	19/25	0/8		< 0.0001
CRP (mg/dl)	202.29 ± 153.53	43.50 ± 10.97	1.87	0.07
Hb (g/dl)	12.21 ± 3.64	13.63 ± 1.46	0.66	0.52
Platelet count (Cells/µl)	247,600 ± 108,940	282,000 ± 24,331	0.54	0.60
Leukocyte count (Cell/mm^3^)	9664 ± 4329	3495 ± 710	2.79	0.01
Lymphocyte count (Cell/mm^3^)	1436 ± 914	2553 ± 1158	1.93	0.07
NF-KB luciferase activity (%)	9.13 ± 24.03	36.70 ± 44.48	2.35	0.03
TLR7 mRNA expression (%)	22.39 ± 35.28	34.70 ± 45.70	0.84	0.41
IgM (g/L)	1.54 ± 1.12	0.66 ± 0,36	1.27	0.24
IgG (g/L)	7.66 ± 3.83	8.07 ± 1.07	0.18	0.87
IgA (g/L)	1.66 ± 0.92	0.86 ± 0.56	1.36	0.22
IgE (g/L)	606.67 ± 590.03	49.73 ± 53.94	1.63	0.18
Provean	− 4.39 ± 2.82	− 3.07 ± 2.37	1.28	0.21
SIFT	0.05 ± 0.07	0.13 ± 0.15	1.83	0.08
PSEP time (my)	440.15 ± 202.83	280.70 ± 130.91	2.25	0.03
SNAP2	14.10 ± 50.21	− 10.20 ± 70.17	1.09	0.28
CADD score	23.49 ± 4.70	20.95 ± 5.37	1.34	0.19
Thermolability	− 1.31 ± 1.94	− 1.26 ± 1.97	0.06	0.95

In order to validate the ssRNA docking data with the experimental data, we have used three ssRNA motifs, i.e., UUUUUU, AAUUAA, and CCUUCC and compared the experimental derived dissociation constants (KD) with ΔG values obtained by docking to TLR7^24^. Except for the GGUUGG motif, the KD values showed direct proportionality with ΔG values (Table 4).

**Table 4:** Validation of ssRNA docking with experimental data.

ssRNA Motif (5′-3′)	KD (nM)	ΔG (Kcal/mol)
UUUUUU	230 ± 30	− 280.44
AAUUAA	320 ± 19	− 378.33
CCUUCC	100 ± 21	− 257.54
GGUUGG	680 ± 160	− 292.49

KD: Dissociation constant; ΔG: Gibbs free energy change associated with Protein–ligand complex formation.

**Figure 2:**
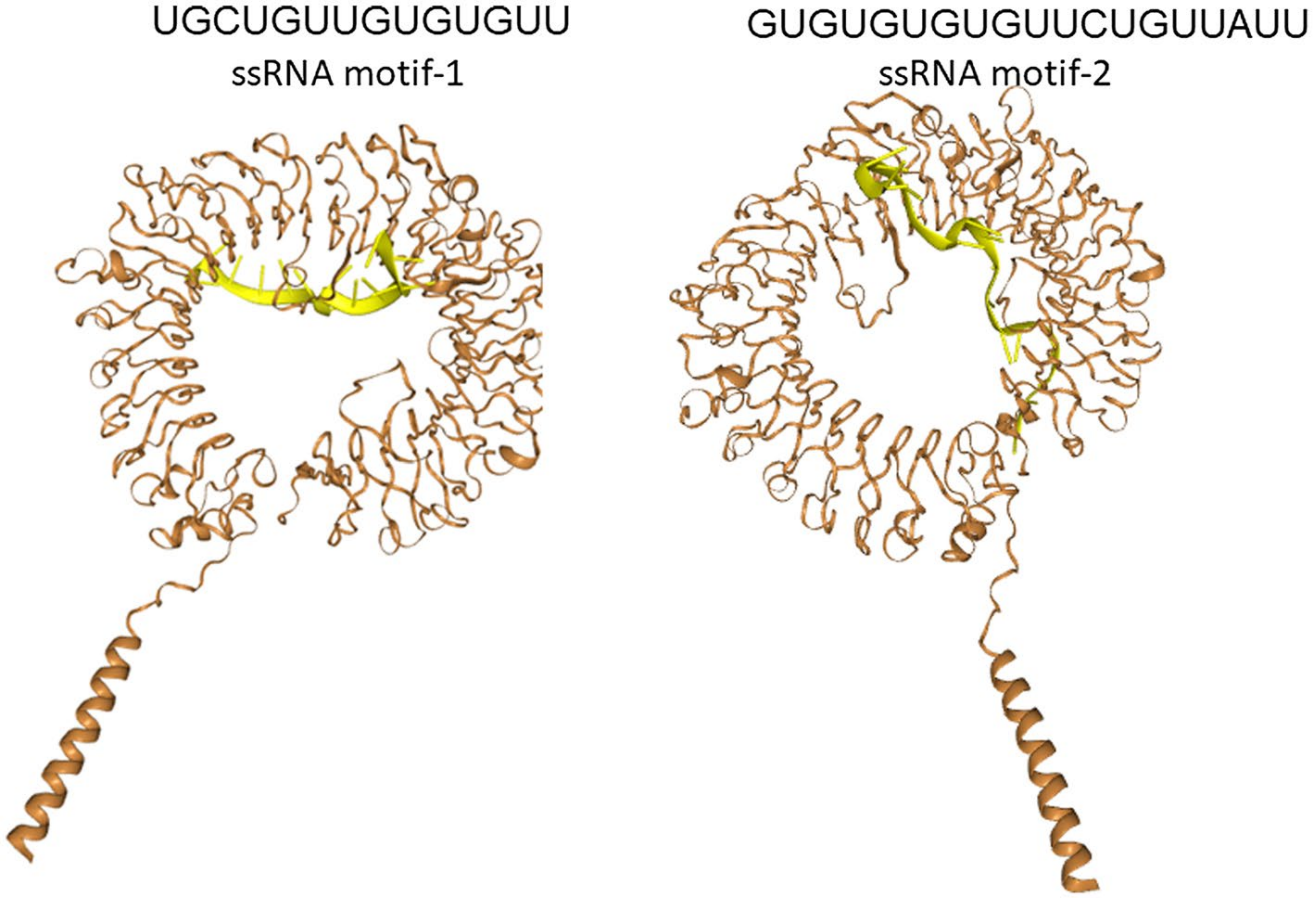
Presence of two ssRNA binding sites in the TLR7. We have used two ssRNA motifs i.e., 5’-UGCUGUUGUGUGUU-3′ and 5′-GUGUGUGUGUUCUGUUAUU-3′ previously reported to induce TLR-MyD88 signaling following COVID-19 infection and docked these variants with the reported TLR7 variants and demonstrated the presence of two binding sites of RNA.

**Figure 3:**
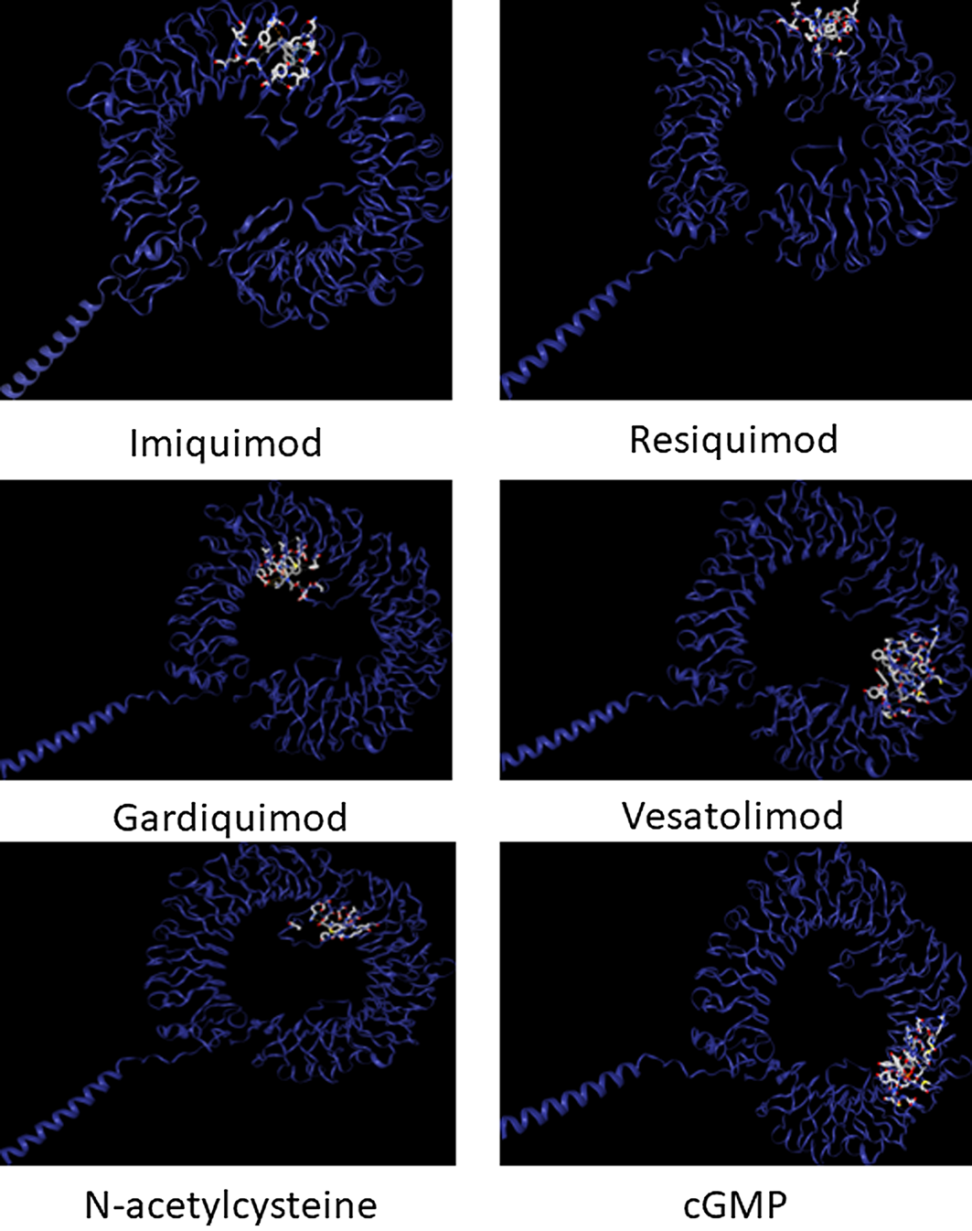
Binding sites of TLR7 agonists, N-acetyl cysteine and Cyclic GMP. Imiquimod, Resiquimod, Gardiquimod, and N-acetylcysteine bind to site 1 while Vesatolimod and cGMP bind to site 2.

We have used two ssRNA motifs i.e., 5′-UGCUGUUGUGUGUU-3′ and 5′-GUGUGUGUGUUCUGUUAUU-3′ previously reported to induce TLR-MyD88 signaling following COVID-19 infection and docked these ssRNAs with the reported TLR7 variants and demonstrated the presence of two binding sites of ssRNA (Fig. 2). The docking studies showed that LOF and hypofunctional variants showed significant impairment in sensing ssRNA motif UGCUGUUGUGUGUU (− 328.66 ± 26.03 vs. − 354.08 ± 27.70, *p* = 0.03) at Site 1 while no such impairment in sensing ssRNA motif GUGUGUGUG-UUCUGUUAUU (− 320.42 ± 42 vs. − 336.41 ± 23.31) at Site 2. We could not perform docking studies on the variants identified in our subjects as they are in the non-coding region (intronic and 3′-UTR). The Q11P variant identified in women showed to have good binding affinity to ssRNA1 (− 371.15 vs. − 363.74 kcal/mol) and ssRNA2 (− 354.37 vs. − 304.76 KCal/mol) compared to that of the wild TLR7 protein. Imiquimod, Resiquimod, Gardiquimod, and N-acetylcysteine bind to site 1 while Vesatolimod and cGMP bind to site 2 (Fig. 3).

The third objective was to evaluate the impact of the TLR7 mutation on the formation of the TLR7-MyD88-TIRAP complex, which is essential for downstream signaling. Asano et al.^5^ have shown that plasma dendritic cells (pDCs) infected with SARS-CoV-2 for 24 h exhibit low IFN-α2 in TLR6 deficient patients compared to their healthy relatives (*p* = 0.03) with significant lowering of Type I/III IFN mRNA (*p* < 0.0001). Van der Sluis highlighted the importance of TLR7-MyD88 pathway in sensing SARS-CoV-2 by exhibiting severe impairment in the induction of type I IFN α and CXCL10 in the MyD88 knockout pDCs on exposure to SARS-CoV-2^25^. Our in silico models corroborate with these studies and demonstrate that deleterious mutations disrupt the protein–protein interactions necessary for TLR7-MyD88-TIRAP complex formation. MyD88 binding showed positive association with TLR7 activity (β: 40.279, SE: 12.182, *p* = 0.003).

**Figure 4:**
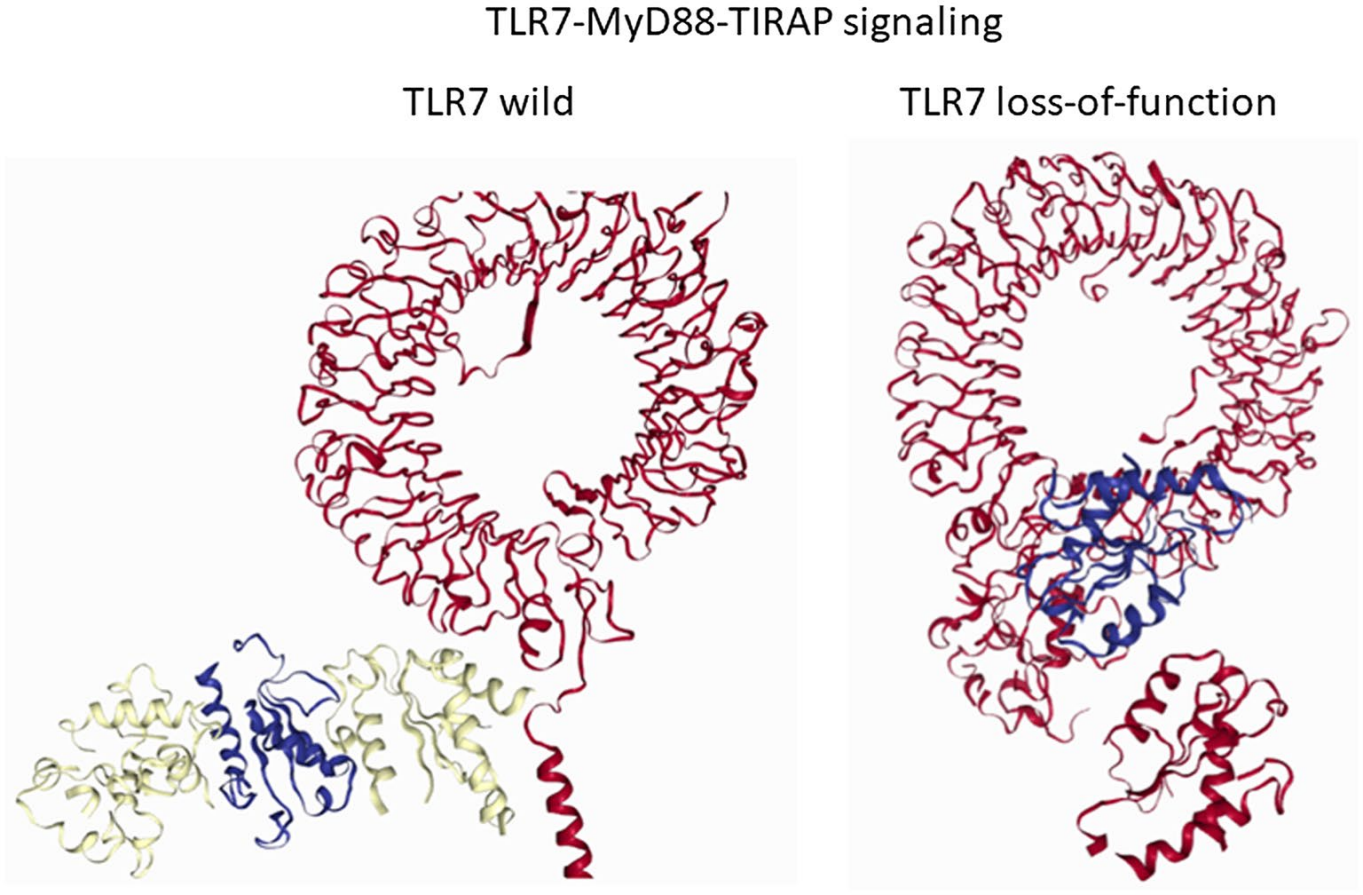
Disruption of TLR7-MyD88-TIRAP complex in loss-of-function (LOF) variants of TLR7. We have studied protein–protein interactions among TLR7, MyD88 and TIRAP using LZerD protein docking server in the presence and absence of TLR7 mutations. As shown in Fig. 1A, TLR7-MyD88-TIRAP complex formation helps in downstream signaling in the absence of TLR7 mutation. In LOF variants of TLR7, downstream signaling is impaired due to disruption of TLR7-MyD88-TIRAP complex as shown in Fig. 1B.

**Figure 5:**
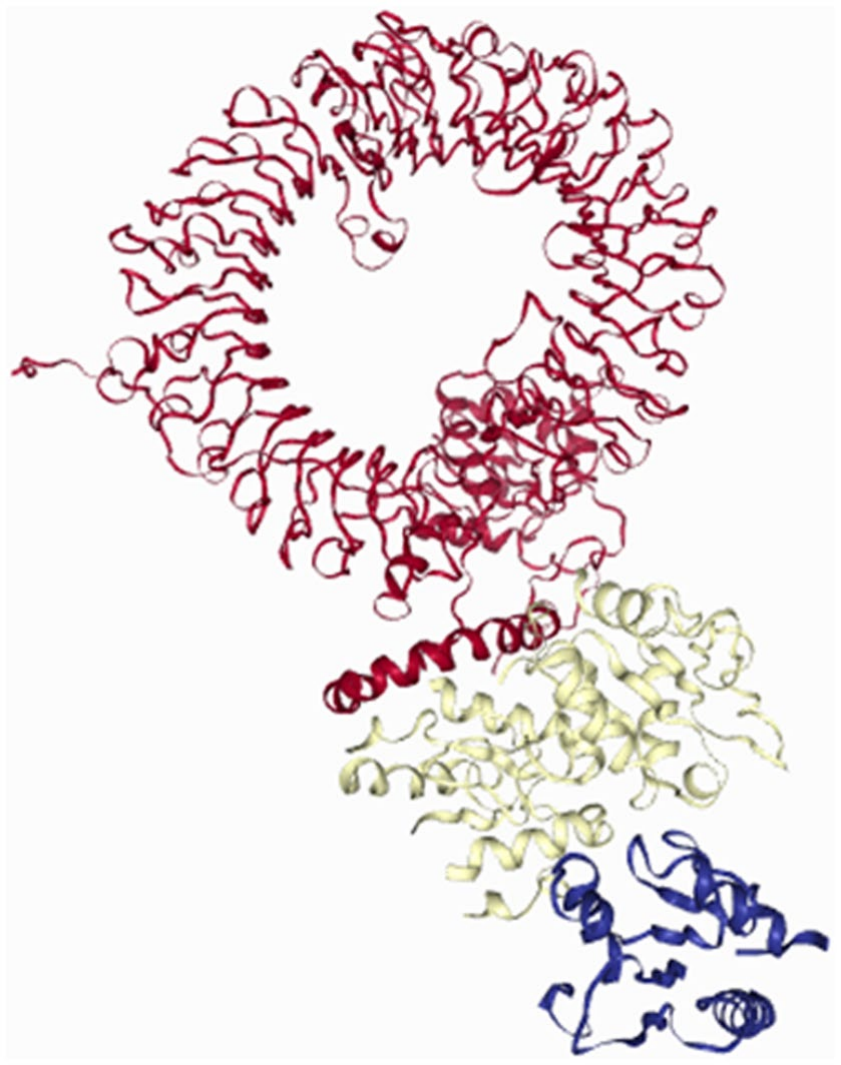
TLR7-MyD88-TIRAP complex in Q11P TLR7 variant. TLR7 Q11P polymorphism showed no significant impact on TLR7-MyD88-TIRAP complex formation.

Disruption in TLR7-MyD88-TIRAP complex was associated in TLR7 mutations with reduced NF-KB luciferase activity (2.47 ± 3.83 vs. 34.00 ± 42.99%, *p* = 0.005). N75H, L134P, N158Tfs11*, D244Y, F310L, I505T, H630Y, I657T, K684*, Q710fs, P715S, and H781L mutations are associated with disruption of this complex (Fig. 4). Although the TLR7-MyD88-TIRAP complex is intact in the presence of the F670Lfs * 8 mutation, none of the TLR7 agonists showed binding affinity to the targeted site. Although the L988S mutation did not disrupting the complex, the binding affinity to imiquimod was altered. Q11P variant observed women has no adverse impact on the formation of the TLR7-Myd88-TIRAP complex. (Fig. 5).

As mentioned in the Table 5, all the TLR7 agonists tested have very lower binding affinity to loss-of-function variants and hence cannot stimulate IFN production. This is further substantiated by the experimental data of the following studies. Van der Made et al.^6^ and Solanich et al.^7^ have demonstrated decreased mRNA expression of IRF5, IFNB1 and ISG15 in the primary peripheral blood mononuclear cells derived from patients with loss-of-function TLR7 mutations (Q710fs, V795F and W933R) upon stimulation with imiquimod. Mantovani et al. demonstrated that imiquimod treatment is effective in hypofunctional and hypomorphic variants (A288V, A448V and V219I) in triggering antiviral response by upregulating antiviral ISGs and proinflammatory cytokine and chemokine genes, which is consistent with our in silico study showing increased binding affinity to hypofunctional and neutral variants^11^. The utility of TLR7 agonists as adjuvants in developing vaccines against different variants of SARS CoV-2 virus demonstrated in several recent studies. Yin et al. used Gardiquimod nanoparticle adjuvant to enhance antibody response to a SARS-CoV-2 subunit vaccine against multiple viral variants^26^. Zhang et al. developed a vaccine by conjugating a TLR7 agonist to S1 subunit of SARS-CoV-2 spike protein, which was effective in inducing neutralizing antibodies against SARS-CoV-2 and all its variant thus boosting humoral and cellular immunity^27^.

**Table 5:** Differences in binding affinities of TLR7 agonists based on TLR7 functionality.

TLR7 agonist	Binding affinity based on functionality			F value	*P* value
	Loss-of-function	Hypofunctional	Neutral		
Imiquimod	− 1.47 ± 3.16	− 5.60 ± 3.75	− 7.38 ± 0.48	9.01	0.001
Resiquimod	− 1.76 ± 3.14	− 5.83 ± 3.93	− 5.74 ± 3.21	4.35	0.026
Gardiquimod	− 1.30 ± 2.80	− 5.80 ± 3.90	− 5.54 ± 3.10	5.98	0.008
Vesatolimod	− 2.16 ± 3.86	− 6.15 ± 4.10	− 6.90 ± 3.86	3.74	0.040

**Figure 6:**
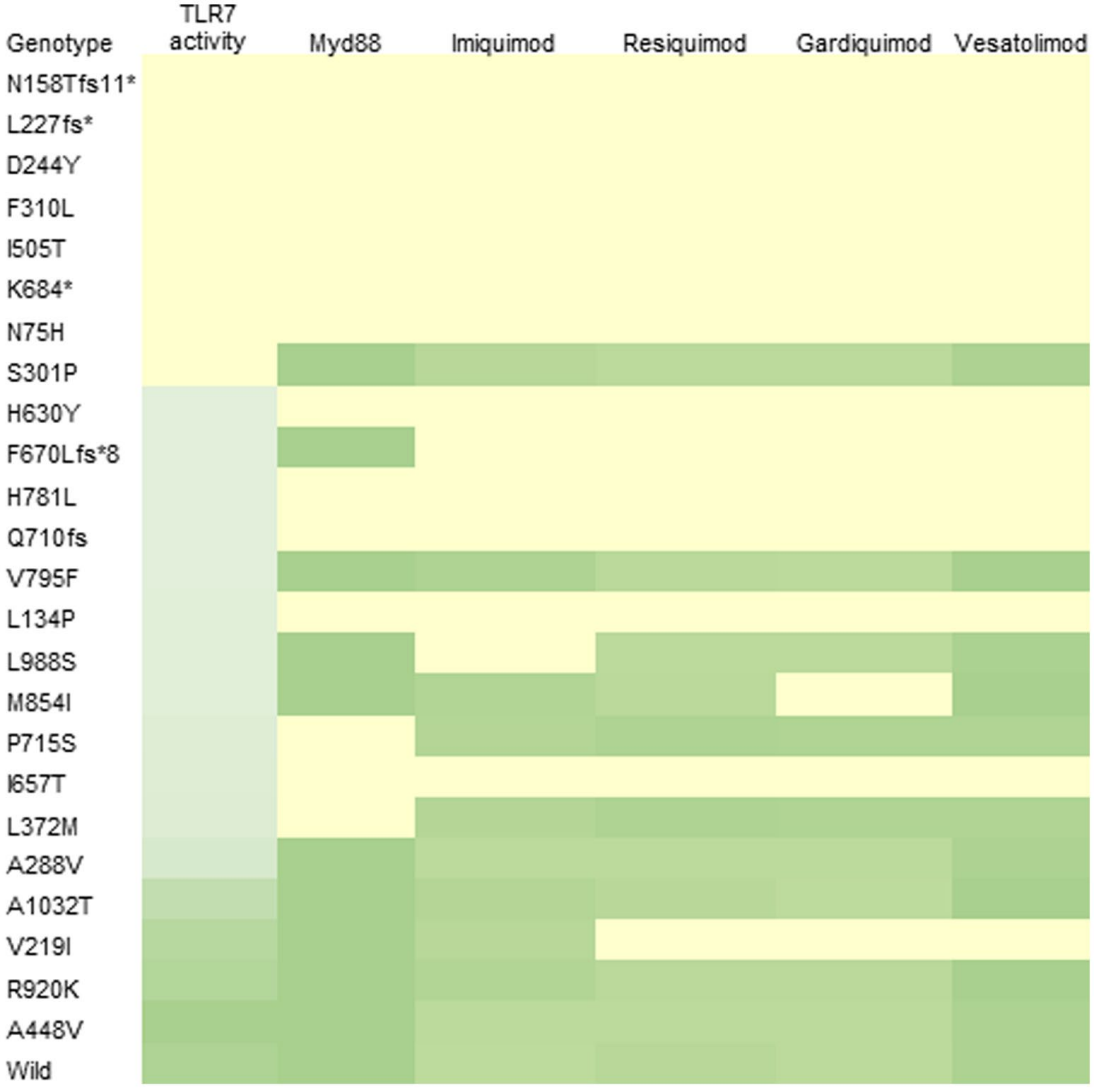
Association of TLR7 activity with MyD88 signaling and agonist binding. The heatmap analysis showed association of Loss-of-function TLR7 mutations with impaired MyD88 signaling and agonist binding. Certain hypofunctional variants are capable to form TLR7-MyD88-TIRAP complex and different TLR7 agonists can effectively bind to them. Neutral/benign variants form TLR7-MyD88-TIRAP complex and different TLR7 agonists can efficiently bind to them.

All the tested TLR7 agonists showed binding affinities towards V795F, S301P, R920K, A1032T, A288V, and A448V mutants thus effective in carrying TLR7-MyD88-TIRAP signaling. Among the TLR7 agonists, only imiquimod showed a binding affinity towards V219I mutant, while none of the other agonists showed no binding. All TLR7 agonists effectively binding to M854I except Gardiquimod and the formation of the TLR7-MyD88-TIRAP complex remains unaffected. Heat map analysis illustrates the association of each mutation with MyD88 signaling and agonist binding (Fig. 6).

Three COVID-19 related deaths were reported in the presence of N158Tfs11*, L988S and Q710Rfs*18 mutations, whose TLR7 activity as measured by NF-KB luciferase activity was 0–2% thus categorizing these as loss-of-function variants. This LOF was shown to disrupt the formation of the TLR7-MyD88-TIRAP complex necessary for downstream signaling associated with the immune response.

**Figure 7:**
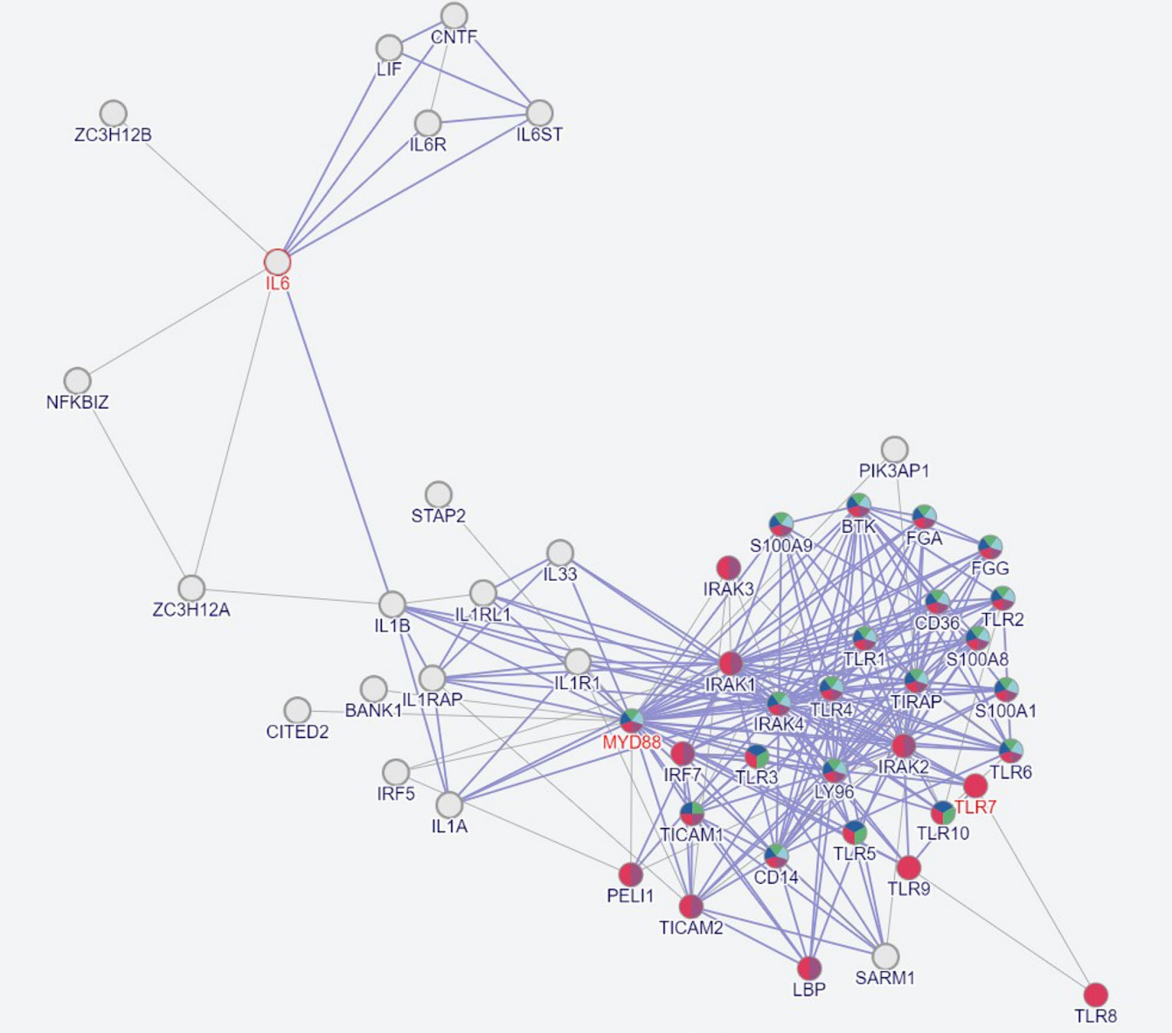
Interactome mapping of TLR7 and MyD88 interactions with 48 different proteins. The interactome map describes 210 interactions among 50 proteins including TLR7/TLR8 and MyD88. This network analysis dictates inflammatory response, TLR-MyD88 signaling pathway, regulation of cytokine production and response to bacteria.

The protein interactome profile showed 210 interactions among 50 proteins. The TLR7 signaling pathway covered TLR2, TLR4, TLR6, TLR8, MyD88, TICAM1, TICAM2, IRAK1, IRAK2, IRAK3, IRAK4 and TIRAP. The TLR7/8 and MyD88 signaling regulate the production of the inflammatory cytokines IL1B and IL6 (Fig. 7).

The TLR7 variants in critical COVID showed a higher affinity for N-acetyl cysteine (NAC) (− 7.68 ± 9.27 vs. − 7.18 ± 1.37, *p* = 0.03). This supports the utility of NAC in critical cases of COVID with LOF variants of TLR7. NAC prevents the activation of NF-κB by scavenging ROS. It inhibits nuclear translocation of IKKb and NF-κB nuclear translocation. NAC also suppresses the synthesis of proinflammatory cytokines^28^. High oral doses of NAC reduced the mortality rate in older men with comorbidities^29^. NAC disrupts the Cys480- Cys488 bridge in the receptor binding domain (RBD) of spike protein^30^. As a result, it prevents virus adhesion to the angiotensin converting enzyme-2 (ACE2) receptor. We used the cryo-electron microscopy structure of TLR7 in complex with the Unc-93 homolog B1 (UNC93B1) as a template^31^. This structure explains the trafficking of TLR from the endoplasmic reticulum to the endosome^31^. The TLR7 transmembrane and juxta membrane regions interact with the N-terminal of UNC93B1^31^. This cryo-electron microscopy structure helped to simulate the different variants of TLR7.

SARS CoV-2 E-protein RNA forms H-bonds with 684LYS, 707SER, 708HIS, 710(B)GLN, 731LYS, 732ASN, 734(B) GLN, 736ARG, 755SER, 756SER, 758LYS, 782HIS, 784(B)ARG residues of TLR7^29^. The residues 627ARG, 736ARG, 758(B)LYS, and 820 (B) HIS form a salt bridge^32^. Interactions of SARS CoV-2 RNA with different variants of TLR7 unravelled RNA sensing ability changes.

Next-generation TLR7/8 adjuvants enhanced vaccine responses in vulnerable populations^33^. Conjugation of p(Mannose-TLR7) to the Spike protein led to higher levels of neutralizing antibodies^33^. Responses to resiquimod adjuvant-induced IFN I and IL-6 in dendritic cell-lymphocyte coculture^34^. Agonists restore the viral sensing ability of TLR7, resulting in an anti-viral response. They induce cellular and humoral immunity, making them ideal adjuvants for vaccines.

SARS-CoV-2 triggers a cytokine storm by activating the IRF3 and NFB transduction pathways^35^. SARS-CoV-2 single-stranded RNA (ssRNA) fragments serve as disease-associated molecular patterns (DAMPs). These DAMPs activate the endosomal TLR7/8 and MyD88 pathways. TLR-MyD88 signaling elicits antiviral responses through IFN, cytokines, and Th1 polarisation^36^. LOF mutations disrupt this pathway.

In the early stages of COVID, circulating plasmacytoid dendritic cells (pDCs) are low. Any perturbation in the detection of viral RNA can lead to severe disease during this phase^37^. The deleterious mutations in TLR7 reduced its mRNA expression^5–8^. This LOF caused a loss of viral RNA sensing ability. Mutations in TLR7 do not affect imiquimod binding. MyD88 showed a higher binding affinity for LOF variants than hypofunctional variants. NAC had a higher binding affinity for TLR7 variants reported to be associated with critical COVID. Therefore, it can restore the antiviral response in TLR7 LOF variants.

In conclusion, TLR7 LOF variants cause COVID-19 severity due to loss of viral RNA sensing ability and MyD88 binding thus disrupting TLR7-MyD88-TIRAP complex formation necessary for downstream signaling. TLR7 agonists can efficiently bind to hypofunctional and neutral TLR7 variants and stimulate TLR7-MyD88-TIRAP mediated signaling to restore anti-viral responses. The agonists that bind to majority of TLR7 variants can serve as effective adjuvants in vaccines. Such agonists should be tested for their efficacy against other viruses also where TLR7-mediated RNA sensing plays a central role in triggering immune response e.g. Filoviruses (Ebola and Marburg).

## Data Availability

The datasets generated and/or analysed during the current study are available in *BioModels Database* MODEL2306170001.
